# Research progress on ferroptosis in diabetic kidney disease

**DOI:** 10.3389/fendo.2022.945976

**Published:** 2022-09-29

**Authors:** You Wu, Yan Chen

**Affiliations:** Department of Endocrinology, the Second Hospital of Jilin University, Changchun, China

**Keywords:** iron-dependent cell death, iron metabolism, diabetic kidney disease, lipid peroxidation, diabetes mellitus

## Abstract

Ferroptosis is a newly discovered form of cell death that differs from other forms of regulated cell death at morphological, biochemical, and genetic levels, and is characterized by iron-dependent accumulation of lipid peroxides. Ferroptosis is closely related to intracellular metabolism of amino acids, lipids, and iron. Hence, its regulation may facilitate disease intervention and treatment. Diabetic kidney disease is one of the most serious complications of diabetes, which leads to serious psychological and economic burdens to patients and society when it progresses to end-stage renal disease. At present, there is no effective treatment for diabetic kidney disease. Ferroptosis has been recently identified in animal models of diabetic kidney disease. Herein, we systematically reviewed the regulatory mechanism of ferroptosis, its association with different forms of cell death, summarized its relationship with diabetic kidney disease, and explored its regulation to intervene with the progression of diabetic kidney disease or as a treatment.

## Introduction

The number of diabetic patients worldwide has more than doubled during the past 20 years (1). About 30-40% of these patients can develop diabetic kidney disease (DKD), of which about 50% can progress to end-stage renal disease (ESRD). DKD is the most common cause of ESRD, and is associated with increased incidence and mortality in diabetic patients. Timely diagnosis and treatment can delay its progression. However, a study by US scholars in 2021 showed that about 50% of patients with type 2 diabetes in CKD 3 stage are undiagnosed (2). In addition, there are differences between the animal models used in the preclinical study of DKD and the clinical studies in terms of age, renal function at the onset of the disease, and combination drugs, which leads to poor predictive value of animal experiments on the results of clinical trials, which increases the difficulty of treatment, so the control of DKD progression is not ideal (3). Current treatments for DKD include controlling blood pressure and blood glucose, and the use of drugs that inhibit the renin-angiotensin system. However, these methods have limited effectiveness in preventing DKD progression. Therefore, deeper understanding of the underlying molecular mechanisms of DKD is needed to develop better therapies. Long-term high blood glucose level induces the expression of advanced glycation end-products, cytokines, growth factors, etc., activates signal transduction pathways, and promotes inflammation, endoplasmic reticulum stress, oxidative stress, mitochondrial dysfunction, and expression of autophagy-related genes, which constitute the main pathogenesis of DKD (4, 5). Ferroptosis is defined as iron-dependent regulated cell death, involving regulation at gene and protein levels, and is associated with abnormal accumulation of lipid reactive oxygen species (ROS), resulting in oxidative stress and cell death (6). Ferroptosis is ubiquitous in the body, and is involved in various physiological and pathological processes. In the pathogenesis of type 2 diabetes, ferroptosis not only leads to insulin secretion disorder, β-cell damage, endoplasmic reticulum stress, and production of ROS but also participates in the development of diabetes-related complications (7). In this review, we discussed the specific mechanism of ferroptosis and its role in DKD.

## Discovery of ferroptosis

In 2003, Dolma et al. found that a novel compound erastin, selectively kills cancer cells differentially expressing RAS compared to other cells. In 2012, Dixon et al. investigated the mechanism by which erastin kills cancer cells using RAS mutations, and formally named this cell death process as “ferroptosis” (8). There are no morphological changes in the cell membrane and chromatin during ferroptosis, which are mainly manifested as decreased mitochondrial volume and mitochondrial crest, and increased mitochondrial membrane density (9). Biochemically, ferroptosis mainly manifests as declined glutathione peroxidase-4 activity, depletion of intracellular glutathione, and increased ROS level. Iron accumulation, glutathione depletion, and lipid peroxidation are indispensable and occur simultaneously during ferroptosis (10).

## Association between ferroptosis and other forms of cell death

Ferroptosis was previously thought to be genetically and biologically different from other forms of cell death, but has been subsequently proven to share a common pathway with these forms (1). Apoptosis: It is now known that reactive oxygen species-induced lipid peroxidation plays an important role in apoptosis, mainly manifesting as lipid peroxidation products, which can be combined with extracellular signal-modulating kinase, p38 and other complexes to activate mitogen-activated protein kinase (MAPK) to activate caspase signal to initiate apoptosis. In addition, protein kinase C (PKC) can also be activated to amplify the apoptosis cascade. Since ferroptosis is accompanied by the formation of ROS and lipid peroxidation products, whether interfering with ferroptosis to reduce the production of ROS and subsequent lipid peroxidation plays a regulatory role in apoptosis needs to be further studied (11). Moreover, a recent study has indicated that ferroptosis-induced endoplasmic reticulum stress is associated with apoptosis. Protein kinase RNA-like endoplasmic kinase (PERK) - eukaryotic initiator 2α (EIF2α) - activating transcription factor 4 (ATF4) pathway-mediated endoplasmic reticulum stress is involved in regulation of enhancer binding protein (C/EBP[CCAAT-enhancer-binding protein] homologous protein] homologous protein, CHOP) and other target genes. Previous studies have shown that CHOP binds to the promoter of the pro-apoptotic protein p53-upregulated apoptotic factor (PUMA) during endoplasmic reticulum stress and induces the expression of PUMA, while trace analysis data have shown that the ferroptosis inducer artesunate (ART) can induce AFT4-dependent gene CHOP expression. In summary, ferroptosis inducers may promote the expression of pro-apoptotic protein PUMA through the PERK-EIF2α-ATF4-CHOP pathway. Interestingly, ART does not induce the expression of other pro-apoptotic proteins such as BCL-2 to promote apoptosis, that is, ferroptosis inducers do not promote apoptosis, which suggests antagonism in the induction of ferroptosis and apoptosis. Further research is needed to understand the role of ferroptosis inducers in PUMA activation for apoptosis (12). Whether a synergistic effect exists between ferroptosis inhibitors and apoptosis remains unknown but is likely based on the common characteristics ROS production and lipid peroxidation. Future research on regulating ferroptosis intervention-related diseases needs to focus on the apoptosis signal transduction pathway. (2) Autophagy: Autophagy is an evolutionarily conserved lysosomal-dependent degradation pathway. Nuclear receptor coactivator 4 (NCOA4)-mediated ferritinophagy can lead to ferroptosis by providing available labile iron. Lipid peroxides in ferroptosis induce autophagy by inhibiting adenosine monophosphate-activated protein kinase (AMPK) activation of mammalian target of rapamycin (mTOR), while knockout of autophagy-related genes such as *Atg5* and *Atg7* can reduce lipid peroxidation and intracellular Fe^2+^ inhibition of ferroptosis (11, 13). Autophagy, lipid peroxides, and ferroptosis involve complex interactions. Inhibition of ferritinophagy can interrupt ferroptosis in metabolic diseases. For example, NCOA4 knockout inhibits erastin-induced ferroptosis, while NCOA4 overexpression may be sufficient for ferroptosis. It is necessary to study the molecular regulatory mechanism of ferritinophagy in diseases (14). A new ferroptosis inhibitor 9a can act on NCOA4 to ameliorate ischemic-reperfusion injury of the nervous system *via* the ferroptosis regulatory pathway, suggesting that NCOA4 is a promising drug target (15). In summary, apoptosis, autophagy and ferroptosis are closely linked by lipid peroxides. Different forms of cell death have unique morphological and biochemical characteristics, but there are some crosstalks between the regulators and components of these processes that jointly regulate cell death, and complete understanding of the interactions between the above processes can provide new insights on ferroptosis-related diseases.

## Regulatory mechanism of ferroptosis

### Induction of ferroptosis

**Figure 1:**
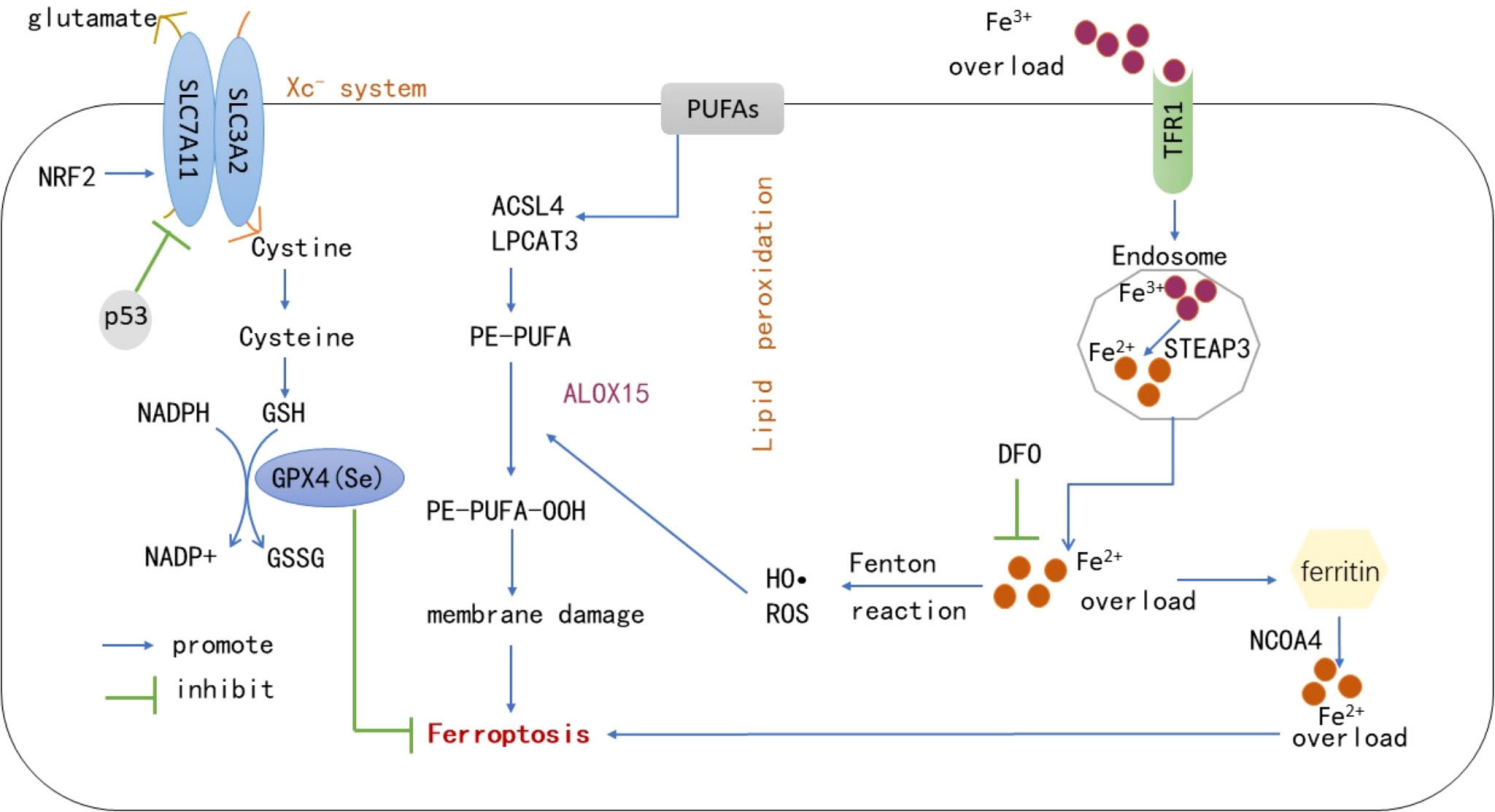
Under the actions of ACSL4, LPACT3 and ALOX15, PUFAs on the cell membrane form PE-PUFA-OOH. Under excessive iron conditions, some iron is stored in the form of ferritin, and the remaining free Fe^2+^ generates numerous ROS and hydroxyl radicals through the Fenton reaction, which induces ferroptosis on the cell membrane. However, GPX4 reduces PE-PUFA-OOH to -OH and inhibits ferroptosis *via* the effect of GSH. The synthesis of GSH is mainly regulated by the Xc^–^system, through which cystine transported into cells is reduced to cysteine to synthesize GSH. p53 can inhibit SLC7A11 expression in this system and promote ferroptosis. In addition, NRF2 can affect the expression of SLC7A11 against ferroptosis. NCOA4-mediated ferritinophagy leads to ferritin production by providing labile iron, deferoxamine can also inhibit ferroptosis by chelating active iron. PUFAs, Polyunsaturated fatty acids; GPX4, Glutathione peroxidase 4; GSH, Reduced glutathione; GSSH, Oxidized glutathione; ACSL4, Acyl-CoA synthetase long-chain family member 4; LPACT3, Lysophosphatidylcholine Acyltransferase 3; ALOX15, Arachidonic acid 15-lipoxygenase; NCOA4, Nuclear receptor coactivator-4; ROS, Reactive oxygen species; TFR1, Transferrin receptor 1; NRF2, Nuclear factor erythroid-related factor 2; DFO, Deferoxamine; STEAP3, six-transmembrane epithelial antigen of the prostate 3.

It is currently believed that the main mechanism of ferroptosis is the catalytic lipid peroxidation of highly expressed unsaturated fatty acids on the cell membrane under the action of unstable Fe^2+^ or lipoxygenase, thereby inducing cell death. In addition, ferroptosis is also manifested by the reduction of the core enzyme GPX4 of the antioxidant glutathione system (1). Role of active iron: Under normal circumstances, the iron entering the body is bound with transferrin in the form of Fe^3+^, enters the cell *via* transferrin receptor 1 on the cell membrane, and is reduced to Fe^2+^ by six-transmembrane epithelial antigen of the prostate 3 (STEAP3). The majority of iron is stored in the form of ferritin, and the minority is transported to a labile iron pool (LIP) in the cytoplasm *via* divalent metal transporter 1 (16). The intracellular iron output is mainly mediated by ferroportin (FPN), the main reason is that during iron overload in the body, a large amount of free iron in the LIP in the cells, i.e., Fe^2+^, can provide hydroxyl radicals through the Fenton reaction and participate in lipid peroxidation, resulting in ferroptosis. The use of iron chelating agents (deferoxamine) to inhibit ferroptosis corroborates the role of iron overload in the process of ferroptosis, so ferroptosis is closely related to the steady state of iron metabolism in the body. Hepcidin can regulate FPN expression and affect the iron level in the system, while iron reaction elements (IREs)/iron regulatory proteins 1, 2 (IRP1, IRP2) regulate iron homeostasis at the cellular level (17). The imbalance of iron intake, storage, utilization, and outflow in the body affects the sensitivity of cells to ferroptosis. In addition to common iron transporters, heat shock proteins, contain iron enzymes such as heme oxygenase-1 (HO-1), which regulate ferroptosis through other pathways such as lipid peroxidation (18). (2) Main processes of lipid peroxidation: The peroxidation of polyunsaturated fatty acids on the cell membrane is the key process of ferroptosis. Polyunsaturated fatty acids first form PE-PUFA under the actions of Acyl-CoA synthetase long-chain family member 4 (ACSL4) and lysophosphatidylcholine acyltransferase 3 (LPACT3), followed by formation of PE-PUFA-OOH through the action of lipoxygenase or non-enzymatically through autoxidation, which ultimately causes cell death (19). Lipid peroxidation mainly occurs in two ways: non-enzymatic radical chain reaction and enzyme catalytic occurrence, non-enzymatic radical chain reaction is mainly mediated by iron provided by hydroxyl radical through Fenton reaction, and enzyme catalytic reaction is mediated by the aloxygenases (ALOXs), especially ALOX15. Lipoxygenase was initially considered as an important driving factor of lipid peroxidation, but its low expression in some cancer cells suggested that other enzymes may mediate lipid peroxidation. Koppula et al. summarized previous studies and concluded that cytochrome P450 reductase transfers electrons from NADPH to oxygen to produce hydrogen peroxide, thereby driving lipid peroxidation, membrane rupture and ferroptosis (20). Therefore, ALOXs may not be necessary for ferroptosis, and may also function in some more complex environments or situations by supplementing the auto-oxidation pathway, which needs to be further studied. Many hypotheses have been proposed on the mechanism by which lipid peroxides cause ferroptosis, including changes in cell membrane structure and permeability affecting cell survival. Lipid peroxides that can break down to produce toxic derivatives such as malondialdehyde (MDA), causing DNA and protein damage. In addition, once lipid peroxides are formed, they may further amplify ROS signaling and drive the mitochondrial cysteine protease signaling pathway, linked to pyroptosis (19). The substrates of ferroptosis are mainly polyunsaturated fatty acids. Recent studies have found that long-chain saturated fatty acids also participate in ferroptosis, but the mechanism remains unclear. Through endogenous metabolites and genome-wide CRISPR screening, Cui et al. confirmed that peroxisomal fatty acyl-CoA reductase 1 (FAR1) is a key factor in ferroptosis mediated by long-chain saturated fatty acids (21). Interestingly, a recent study also reported that exogenous monounsaturated fatty acids suppress ferroptosis requiring acyl-CoA synthetase long-chain family member 3 (ACSL3), which is related to the inhibition of lipid ROS accumulation and reduction of phospholipid levels of oxidizable polyunsaturated fatty acids (22). Thus, the activation of ACSL4 rather than other homologous enzymes is necessary for lipid peroxidation, and ACSL4 expression can regulate the occurrence of ferroptosis. (3) The collapse of the antioxidant system: 1) Xc^-^system-GSH-GPX4: GPX4 is a key defense mechanism to prevent cellular ferroptosis, with selenocysteine in its active center. It reduces toxic lipid hydroperoxides to corresponding hydroxyl derivatives to inhibit ferroptosis *via* intracellular glutathione (23). Lipid peroxidation and subsequent ferroptosis were observed in mice with GPX4 conditional knockout, indicating that GPX4 is a key regulator of ferroptosis (24). GSH acts as an electron donor during GPX4 involvement in ferroptosis, so regulation of the GSH axis is necessary to maintain GPX4 activity. Cystine, one of the components of GSH, is the main limiting process of GSH synthesis. It is transferred into cells by the cystine/glutamate reverse transporter (Xc^–^system), which is composed of SLC7A11 and SLC3A2. Inhibition of this system can lead to depletion of cysteine in cells, and induce ferroptosis (25). The nuclear factor erythroid-related factor 2 (Nrf2) affects SLC7A11 expression to resist ferroptosis, and p53 protein also regulates its expression to affect cysteine intake, thereby blocking GSH synthesis and inducing ferroptosis (26). The active center of GSH contains selenocysteine, so the regulation of the selenium axis also affects the activity of GPX4. The mechanism of synthesis of GPX4 is not well understood. Zhang et al. found that cystine and cysteine promote GPX4 protein synthesis by activating rapamycin complex 1 (mTORC1), and its inactivation sensitizes cancer cells to ferroptosis by reducing GPX4 (27). These results suggested that the synthesis of GSH can be regulated by regulating the Xc^–^system to affect ferroptosis. In addition, some cells can also synthesize cysteine from methionine *via* the transsulfuration pathway, which resists ferroptosis to some extent (28). Glutamate-cysteine ligase activity has been found to prevent the accumulation of glutamate in cells under cysteine deficiency, thereby preventing ferroptosis in non-small cell lung cancer (29). 2) Nuclear factor erythroid-related factor 2 signal pathway: NRF2 is involved in constituting and controlling defense pathways for oxidative stress and may play a role in regulating ferroptosis, given that many proteins stored and transported with iron are controlled by NRF2, which also affects enzymes associated with GSH synthesis as described above, so targeting NRF2 to regulate lipid peroxidation and ferroptosis is a viable disease intervention strategy (30, 31). Therefore, GPX4 inactivation is not the only condition for ferroptosis, and the collapse of many antioxidant mechanisms in the body leads to the occurrence of ferroptosis. In summary, ferroptosis is the result of a comprehensive process, which requires the high expression of lipid-promoting peroxidases such as ACSL4, the role of active iron, and the collapse of antioxidant systems such as GPX4. The key processes involved in ferroptosis are shown in **Figure 1**.

### Inhibition of ferroptosis

Numerous mechanisms in the body inhibit ferroptosis: (1) NADPH-FSP1-coenzyme Q10 pathway: GPX4 is considered as a major antioxidant of ferroptosis. Different cancer cells were found to have different sensitivities to GPX4 inhibitors, and it was speculated that there were other factors controlling resistance to ferroptosis. In 2019, ferroptosis suppressor protein 1 (FSP1) was identified as an important antioxidant protein, which acts through coenzyme Q10 (ubiquinone). The reduced form of ubiquinone can capture active free radicals and reduce the production of intracellular lipid peroxides. Moreover, FSP1 can catalyze the regeneration of coenzyme Q10 through NADPH (32). As the main effector of the FSP1 pathway, supplementation with selenium and coenzyme Q10 has been reported to reduce cardiovascular mortality in the elderly (33). In summary, NADPH-FSP1-CoQ10 exists as a parallel system independent of GPX4 action, and inhibition of FSP1 may be an effective strategy to promote death of cancer cells and other diseases. (2) GCH-1-BH4: Tetrahydrobiopterin has antioxidant effects *in vitro*, and its role in regulating ferroptosis has been recently clarified. Tetrahydrobiopterin can reduce lipid peroxidation by producing coenzyme Q10 to reduce oxidative damage and cause lipid remodeling (34). GTP cyclohydrolase 1 (GCH-1) is a rate-limiting enzyme regulating the synthesis of tetrahydrobiopterin. Overexpression of GCH-1 has a protective effect on RSL3-induced ferroptosis, but not on apoptotic inducers, indicating that GCH-1 selectively protects cells against ferroptosis (35) (3). Post-translational modifications (PTMs): PTMs include phosphorylation, acetylation, methylation, etc. Most PTMs are reversible. PTMs not only diversify the function of proteins but also enable cells or organisms to respond quickly and strictly to stress. The role of PTMs in ferroptosis has gradually become a research hotspot in recent years (35). Recent studies on tumor cells reported that when cells are hungry, energy stress activates the AMP-activated protein kinase (AMPK), activates of acetyl-CoA carboxylase (ACC) phosphorylation, and further inhibits the synthesis of polyunsaturated fatty acids and ferroptosis. Activating this process has been found to prevent renal ischemia-reperfusion damage. AMPK can promote ferroptosis by inhibiting the transport of SLC7A11-mediated cystine, although its role in ferroptosis remains controversial. Thus, AMPK and ferroptosis need further study (35–37). The role of AMPK in ferroptosis is related to phosphorylation. There are few studies on how other PTMs are involved in the regulation of ferroptosis. Elucidating PTMs associated with inhibition of ferroptosis under different conditions is an interesting research direction for the future.

## Ferroptosis and DKD

With the increase in global prevalence of diabetes, the prevalence of chronic kidney disease caused by type 2 diabetes has increased from 1.39% in 1999 to 1.52% in 2009 and 1.74% in 2019 (38). Typically, proteinuria is considered as a biomarker of DKD, preceding the loss of renal function. However, a subset of patients have no proteinuria but develop loss of renal function, which is also known as non-proteinuria diabetic nephropathy, which indicates that DKD has clinical heterogeneity and increases the difficulty of treatment (39). The pathogenesis of traditional DKD is believed to be caused by changes in renal hemodynamics (high stress, high filtration, high perfusion), increased oxidative stress caused by ischemia and abnormal glucose metabolism, inflammation, and hyperactivity of the renin-angiotensin-aldosterone system. Recent molecular and cellular studies have continued to explore new areas of DKD pathogenesis, including genetic and epigenetic modifications, podocyte autophagy, and mitochondrial dysfunction, providing more possible directions for the treatment of DKD (40). The role of ferroptosis was first identified in renal ischemic-reperfusion injury, and there are limited studies on DKD. Previous studies found that iron-chelating agents could delay the progression of DKD, the underlying mechanism may be that iron chelating agents exert a protective renal effect by reducing oxidative stress, inflammation, and tubular interstitial fibrosis. However, the exact mechanism by which excessive iron promotes DKD progression remains unclear (41, 42). Because ferroptosis process is accompanied by excessive lipid ROS production, which can lead to oxidative stress, and kidney-rich mitochondrial structure is more vulnerable to oxidative stress damage, the traditional pathogenesis of DKD is also involved in oxidative stress, which suggests that ferroptosis may be associated with DKD. Many scholars have also aimed to explore new ways to control the progression of DKD based on this perspective. The mechanism of ferroptosis in DKD was initially studied mainly at the cellular and animal levels. For example, Wang et al. explored the role of ferroptosis in the progression of DKD using *in vivo* and *in vitro* experiments, and found that ferroptosis-related protein GPX4 expression was decreased, ACSL4 expression was increased, and lipid peroxide products and iron content were also increased in mouse models of DKD (43), which was similar to the results of Li et al. who also found that Nrf2 levels were decreased in the DKD animal model, which inhibited ferroptosis by upregulating Nrf2 through fenofibrate therapy and delayed the progression of DKD in mice (41), revealing the development mechanism of DKD from a new perspective. As mentioned above, the occurrence of ferroptosis is related to the NRF2 signaling pathway. Therefore, Li’s research links ferroptosis more closely with DKD. Subsequently, Kim et al. also reported that ferroptosis was associated with DKD. They evaluated changes in ferroptosis-related molecules in renal biopsy tissues of patients with DKD, and found that SLC7A11 and GPX4 mRNA expression was reduced in renal tubules (44). The above studies have confirmed that ferroptosis is associated with DKD, but the mechanism is unclear. Feng et al. found that ferroptosis can damage renal tubules through hypoxia-inducible factor-1α (HIF-1α)/heme oxygenase (HO-1) pathway, while the selective ferroptosis inhibitor Ferostatin-1 (Fer-1) treatment inhibits the expression of HIF-1α and HO-1, and reduces tubular damage and fibrosis in diabetic mice by reducing tubular iron overload, inhibiting ROS formation, oxidative stress, and lipid peroxidation (45). The association between ferroptosis and DKD was also studied at the clinical level. We found that the expression level of ferroptosis-related protein GPX4 was reduced in the serum of patients with DKD, while the expression of ACSL4, PTGS2, HMGB1, ROS release and MDA generation were upregulated. The inhibition of HMGB1 was further found to promote the expression of Nrf2 to prevent glucose-induced mesangial cell ferroptosis and inhibit the inflammatory response. These findings provide new treatment strategies for DKD by HMGB1 and ferroptosis (46). High mobility group box 1 (HMGB1) is a typical damage-associated molecular pattern (DAMP), which are endogenous mediators causing inflammation, and can be released by apoptosis, ferroptosis, and necrosis (47). Once released, HMGB1 can further bind to receptors such as Toll-like receptor 4 (TLR4) and glycosylation end-product specific receptor (AGER) to mediate immune responses. Therefore, inhibition of HMGB1 release and extracellular activity is a potential anti-inflammatory strategy for the treatment of diseases such as DKD (48). In summary, the above studies have shown that ferroptosis also plays a pathological role in the development of DKD. The main studies on ferroptosis with DKD are listed in **Table 1**.

**Table 1:** Mechanism and biochemical features of ferroptosis in DKD.

Cell/Animals/clinical	Mechanism	Biochemical features	Reference
Animal: STZ-induced diabetic mice and db/db mice Cell: NRK-52E cells and HK-2 cells	ACSL4 regulates ferroptosis	Increase in ACSL4 and MDA Increase in ACSL4	Wang Y, et al. (43)
Cell: NRK-52E cells Animal: STZ-induced diabetic mice Clinical: Kidney biopsy samples	N.A.	Decrease in xCT, GPX4 and GSH Increase in MDA, 4-HNE, iron and FTH1 Decrease in xCT and GPX4	Kim S, et al. (44)
Animal: db/db mice	HIF-1α/HO-1 pathway might be regulated ferroptosis	Decrease in GSH-Px, CAT, SOD Elevated ferritin, HIF-1α/HO-1 and increase in MDA	Feng XM, et al. (45)
Cell: Renal mesangial SV40-MES 13 cells Clinical: blood samples collected from DKD patients	HMGB1/Nrf2 regulates HG induced ferroptosis	Decreased GPX4 Increase in ROS, MDA, ACSL4, PTGS2 and LDH release	Wu Y, et al. (46)

N.A., GPX4 regulates ferroptosis.

## Regulation of ferroptosis for the treatment of DKD

Ferroptosis is related to the action of active iron, lipid peroxidation, and weakened antioxidant capacity, and intervention of these processes may inhibit ferroptosis for therapeutic purposes. Current evidence indicates the following: 1) The ACSL4 inhibitor rosiglitazone reduces renal pathological damage in DKD mice by reducing lipid peroxidation (43). 2) Iron chelating agents are also an effective method of inhibiting ferroptosis by reducing excess intracellular iron, since the occurrence of ferroptosis depends on excess intracellular iron producing large amounts of ROS through the Fenton reaction. Kim and Feng et al. have demonstrated that Fer-1 mitigates kidney damage in mice with DKD (44, 45). Since the regulation of ferroptosis is a multi-pathway, more targets can be identified for the prevention and control of DKD. We present several possibilities: 1) Reduce oxidative stress: the production of numerous ROS in cells and the subsequent generation of active free radicals are the key factors mediating lipid peroxidation, and the NADPH-FSP1-CoQ10 pathway in the regulatory mechanism of ferroptosis is independent of the antioxidant mechanism existing in GPX4, mainly by reducing free radicals. Further research is needed to inform whether vitamin E supplementation can inhibit ferroptosis and reduce kidney damage to some extent by capturing active free radicals. 2) NRF2 signaling pathway: NRF2 is known to be one of the defense pathways for oxidative stress *in vivo*, which can neutralize ROS, regulate enzymes involved in iron metabolism and GSH synthesis in ferroptosis. However, Nrf2 overactivation was found to induce ferroptosis through the HO-1 pathway in cancer (49), which is a double-edged sword, so it is necessary to further understand the relationship between the upstream and downstream regulation of Nrf2 and ferroptosis, and mechanisms of alteration of Nrf2 levels in physiological and pathological states. 3) Inflammatory pathway: DKD is widely regarded as a chronic inflammatory disease. The ferroptosis process is accompanied by DAMPs and inflammatory factors changes. DAMPs lead to renal inflammatory cell infiltration through the immune response, and release inflammatory factors, amplify the immune response, resulting in a sustained inflammatory response, related to the progression of DKD. Ferroptosis is complemented by inflammation. For example, in mouse models of nonalcoholic steatohepatitis, ferroptosis has been found to occur with the expression of pro-inflammatory factors such as tumor necrosis factor-α and IL-6, and treatment with deferoxidamine can significantly inhibit the progression of nonalcoholic steatohepatitis, manifested by decreased lipid peroxidation levels (50). In addition, some inflammatory cytokines have also been shown to affect the activity of GPX4 in cancer cells (51), and some anti-inflammatory drugs have been found to inhibit ferroptosis in some cellular models (52). These findings suggest that controlling inflammation during ferroptosis may have a wide range of regulatory effects with clinical benefits. 4) Protein post-translational modification: The changes of various protein activities in the process of ferroptosis may be related to PTMs. Identifying DKD-specific biomarkers to activate or inactivate the protein modifications may more easily regulate ferroptosis for therapeutic purposes. Although the main mechanism of ferroptosis is currently well understood, deeper understanding is needed to provide a better theoretical basis for the treatment of DKD and other diseases.

## Conclusion

When DKD progresses to end-stage renal disease, renal replacement therapy is the only option, which also increases the risk of death, so early intervention is the key. However, the previous drugs used to treat DKD (RAAS inhibitors) have not been found to prevent the occurrence of DKD. With the advances in the pathogenesis of DKD, new drugs for the prevention and treatment of DKD, such as endothelin receptor antagonists, protein kinase C inhibitors, phosphodiesterase inhibitors, etc., are being investigated. The discovery of ferroptosis provides a new approach. However, as mentioned earlier, although ferroptosis has different morphological and biochemical characteristics from apoptosis and autophagy, it is closely linked by lipid peroxidation, and the interaction between these processes should be noted when targeting ferroptosis in the treatment of DKD, whether it is synergistic or antagonistic. Notably, the association between ferroptosis and DKD is mainly verified at the cellular level and in animal models, without any clinical trials. However, understanding the specific mechanism of ferroptosis may provide a strategy for the treatment of DKD. Finally, ferroptosis-specific biomarkers can also be investigated in the future to detect the progression of DKD.

## Author contributions

YW wrote the original draft. YW and YC reviewed and edited the manuscript. All authors contributed to the article and approved the submitted version.

## Funding

This work was supported by the Youth of National Natural Science Foundation of China [Grant Number 82001480].

## Conflict of interest

The authors declare that the research was conducted in the absence of any commercial or financial relationships that could be construed as a potential conflict of interest.

## Publisher’s note

All claims expressed in this article are solely those of the authors and do not necessarily represent those of their affiliated organizations, or those of the publisher, the editors and the reviewers. Any product that may be evaluated in this article, or claim that may be made by its manufacturer, is not guaranteed or endorsed by the publisher.
